# Supplementary material for: Transcriptional heterogeneity of stemness phenotypes in the ovarian epithelium
Source: Commun Biol. 2021 May 5;4:527. doi: 10.1038/s42003-021-02045-w (PMC8100130; doi:10.1038/s42003-021-02045-w)
Supplement: Supplementary file 1 — Description of Additional Supplementary Files [file 42003_2021_2045_MOESM1_ESM.pdf]

Description of Additional Supplementary Files

Supplementary Data 1. Differential expression results for mOSE spheroids compared to monolayer culture

Supplementary Data 2. GSEA results for mOSE spheroids compared to monolayer culture.

Supplementary Data 3. Differential expression results for TGFB1-treated mOSE compared to untreated samples.

Supplementary Data 4. GSEA results for TGFB1 treated mOSE compared to untreated cultures.

Supplementary Data 5. Differential expression results for *Snai1*-overexpressing mOSE compared to wild-type.

Supplementary Data 6. GSEA results for *Snai1*-overexpressing mOSE compared to wild-type.

Supplementary Data 7. Differential expression results for BRCA1-deleted mOSE compared to wild-type.

Supplementary Data 8. GSEA results for BRCA1-deleted mOSE compared to wild-type.
