# Supplementary material for: Transcriptional heterogeneity of stemness phenotypes in the ovarian epithelium
Source: Commun Biol. 2021 May 5;4:527. doi: 10.1038/s42003-021-02045-w (PMC8100130; doi:10.1038/s42003-021-02045-w)
Supplement: Supplementary file 12 — Supplementary Information [file 42003_2021_2045_MOESM12_ESM.pdf]

Supplemental Table 1. qPCR primer sequences

Aldh1a1	Mouse	Forward - ACC CAG TTC TCT TCC ATT TCC
		Reverse - CAT CAC TGT GTC ATC TGC TCT
Cd44	Mouse	Forward - CAC CAT TTC CTG AGA CTT GCT
		Reverse - TCT GAT TCT TGC CGT CTG C
Lgr5	Mouse	Forward - GTC AAA GCA TTT CCA GCA AGA
		Reverse - CTC CAA CCT CAG CGT CTT C
Ly6a	Mouse	Forward - GAC CCT GGA GGC ACA CAG CC
		Reverse - CAT GTG GGA ACA TTG CAG GAC CCC
Nanog	Mouse	Forward - CAG AAG GGC TCA GCA CCA G
		Reverse - AGG CTT CCA GAT GCG TTC A

Supplemental Table 2. Antibodies used for Western blots

Gene	Primary	Secondary
CD44	Rabbit anti-CD44 (Abcam, ab41478)	Donkey anti-Rabbit IgG HRP conjugated (GE HEalthcare Life Sciences, NA934)
	1ug/mL in 5% non-fat milk overnight, 4C	1:5,000 in 5 % non-fat milk 1 hr, room temperature
BRCA1	Rabbit anti-BRCA1 (Santa Cruz Biotechnology, C-20)	Donkey anti-Rabbit IgG HRP conjugated (GE Healthcare Life Sciences, NA934)
	1:250 in 5% non-fat milk, overnight, 4C	1:10,000 in 5% non-fat milk 1hr, room temperature
B-actin	Mouse anti-β-Actin (Sigma, A2228)	Goat anti-Mouse IgG HRP conjugated (Abcam, ab6728)
	1:80,000 in 5 % non-fat milk 1 hr, room temperature	1:20,000 in 5 % non-fat milk 1 hr, room temperature

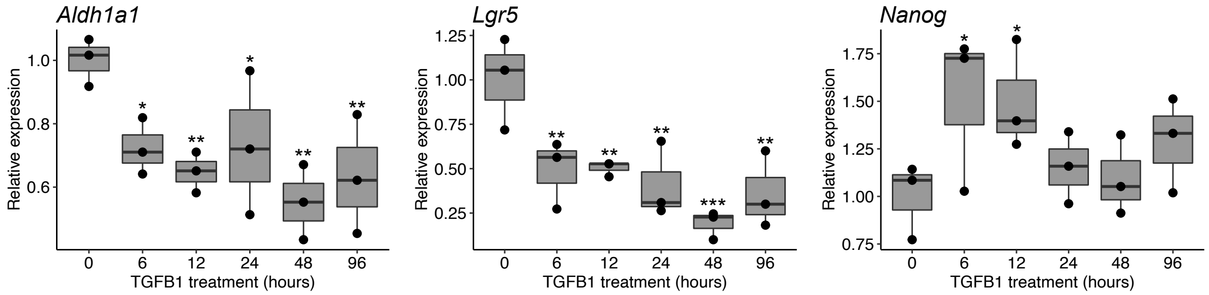

Supplemental Figure 1. Expression of stemness markers through a time course of TGFB1 treatment. qPCR results showing expression of stemness genes following varying lengths of TGFB1 treatment. Boxplots show median value (horizontal black line), estimated 25th and 75th percentiles, and whiskers represent 1.5 times the interquartile range. P-values were computed from the t statistic of a linear regression model. * $p<0.05$, ** $p<0.01$, *** $p<0.001$.

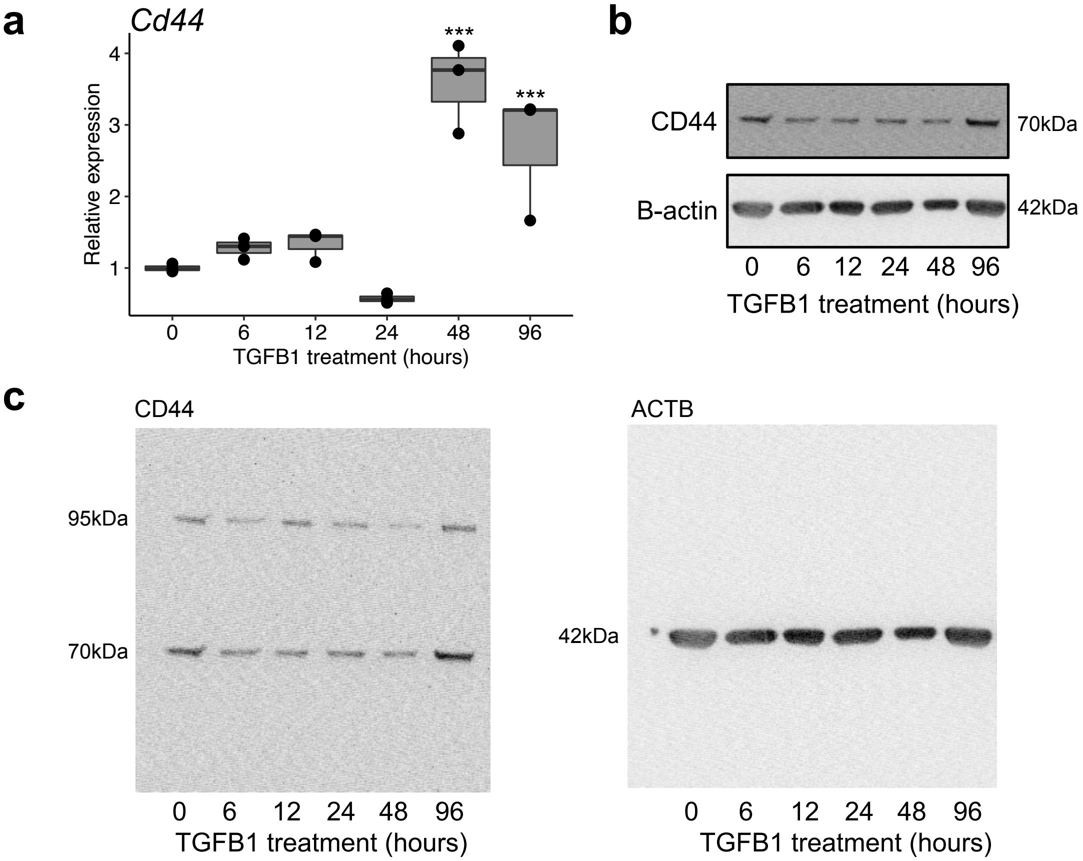

Supplemental Figure 2. CD44 increases following TGFB1 treatment. a. qPCR results showing expression of Cd44 following varying periods of TGFB1 treatment. Boxplots show median value (horizontal black line), estimated 25th and 75th percentiles, and whiskers represent 1.5 times the interquartile range. P-values were computed from the t statistic of a linear regression model. * $p<0.05$, ** $p<0.01$, *** $p<0.001$. **b.** Representative western blot of CD44 and B-actin following following varying lengths of TGFB1 treatment. Densitometric quantifications of three blots are included in Figure 1e. **c.** Uncropped blots of CD44 and B-actin following varying lengths of TGFB1 treatment. Protein detection appears at the predicted molecular weights for each protein.

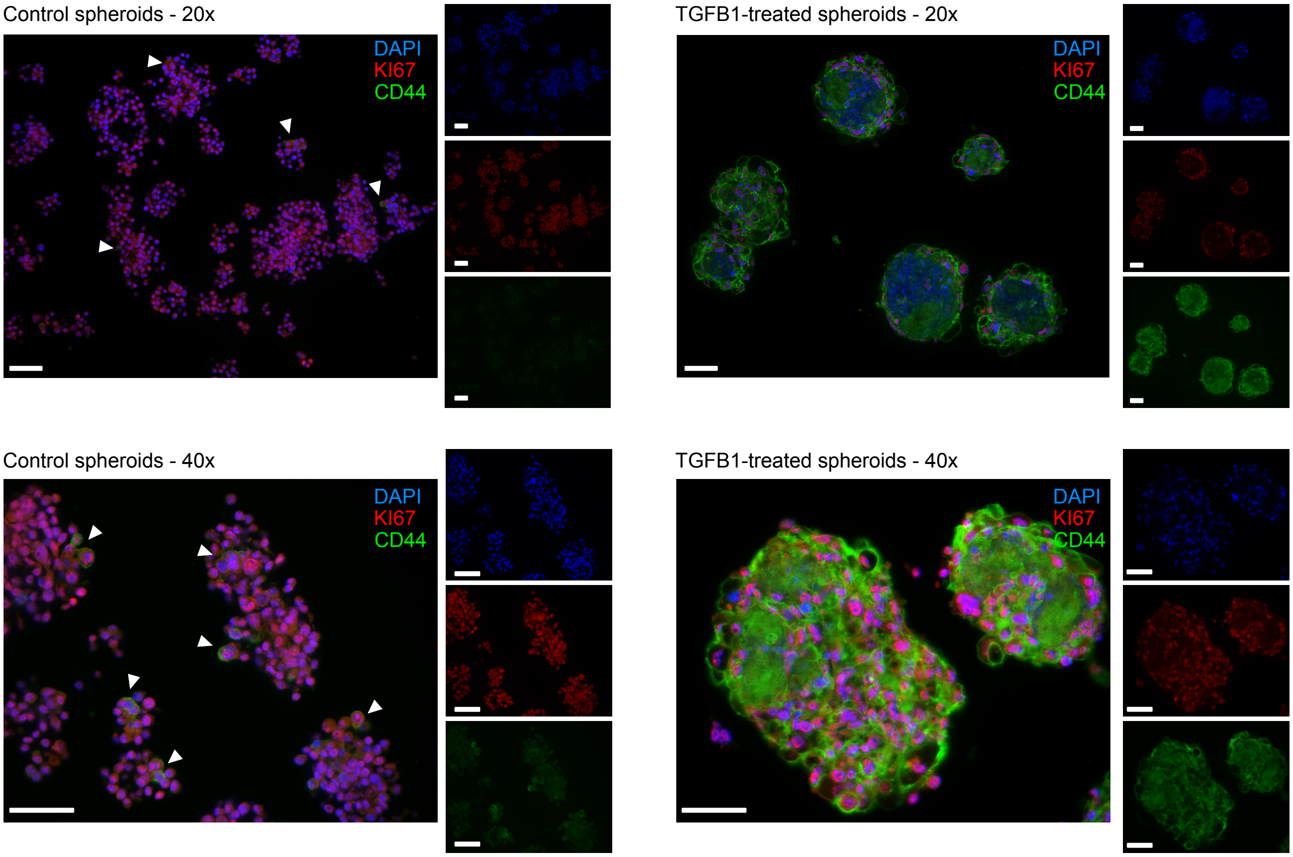

Supplemental Figure 3. Immunofluorescence co-staining of KI67 and CD44 of spheroids from control and TGFB1-treated mOSE. White arrows highlight CD44+ cells in control spheroids. Spheroids were cultured for 14 days. Scale bar = 50µm.

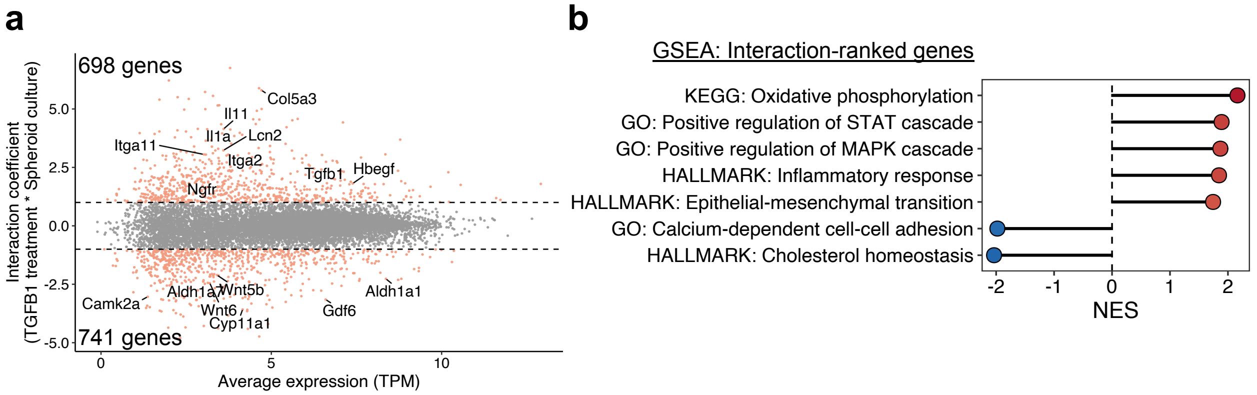

Supplementary Figure 4. a. Plot showing genes with interaction-associated gene expression in TGFB1-treated spheroids. The y-axis represents the interaction coefficient of the differential expression model, with horizontal lines drawn at -1 and 1. Genes with strong interaction effects are those whose expression cannot be explained simply as additive effects of TGFB1 treatment and spheroid culture. Several selected genes are highlighted. **b.** Normalized enrichment scores (NES) of gene sets from GSEA on interaction-ranked genes. High NES values correspond to gene sets associated with genes that have higher expression in TGFB1-treated spheroids than expected from the two experimental variables alone.

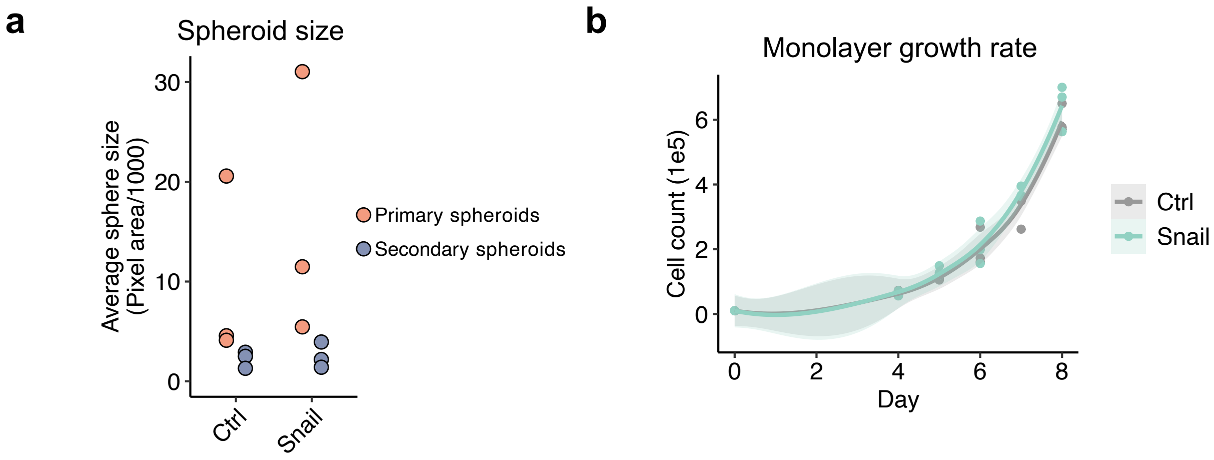

Supplementary Figure 5. a. Relative sizes of spheroids from control (inducible GFP) and Snail-overexpressing mOSE (n=3). Each point on the plot represents the average size of spheroids from 4 fields of view for 4 separate wells. **b.** Proliferation rates of control and Snail-overexpression mOSE (n=3). Each point represents cell counts of an individual replicate and the trend line represents a LOESS regression for each condition, with the shaded area representing the 95% confidence interval.

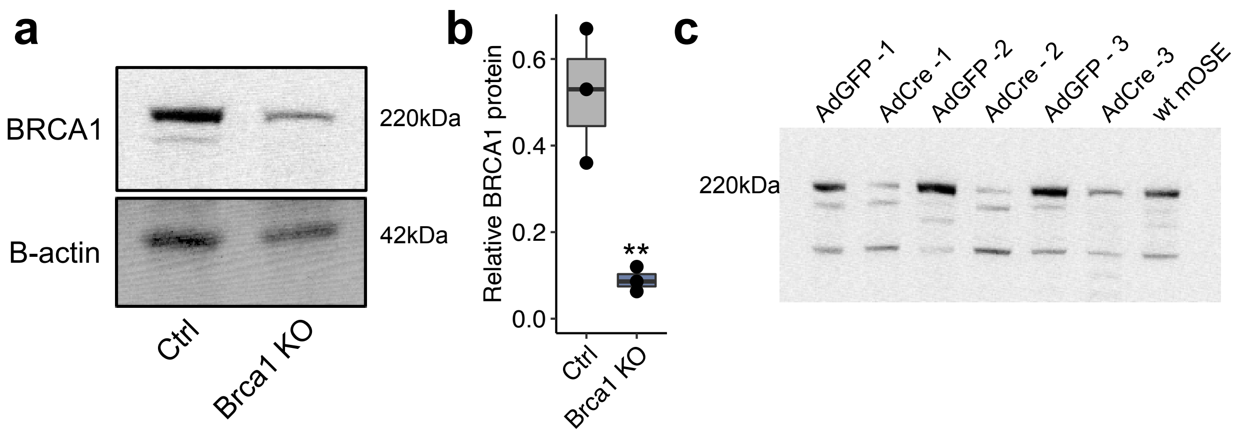

Supplemental Figure 6. Brca1 is efficiently deleted following the expression of Cre recombinase. a. Western blot of BRCA1 and B-actin of Brca1fl/fl mOSE cells following infection with either Ad-GFP (Ctrl) or Ad-Cre (Brca1 KO). **b.** Boxplot of relative BRCA1 protein quantifications from pixel densitometry. N=3 independent infections with Ad-GFP or Ad-Cre. Boxplots show median value (horizontal black line), estimated 25th and 75th percentiles, and whiskers represent 1.5 times the interquartile range. P-values were computed from the t statistic of a linear regression model. ** $p<0.01$. **c.** Uncropped Western blot showing BRCA1 deletion in cells infected with AdCre. Strong protein detection is present at the predicted molecular weight of BRCA1. Wild type mOSE lacking floxed BRCA1 alleles was used as a positive control.

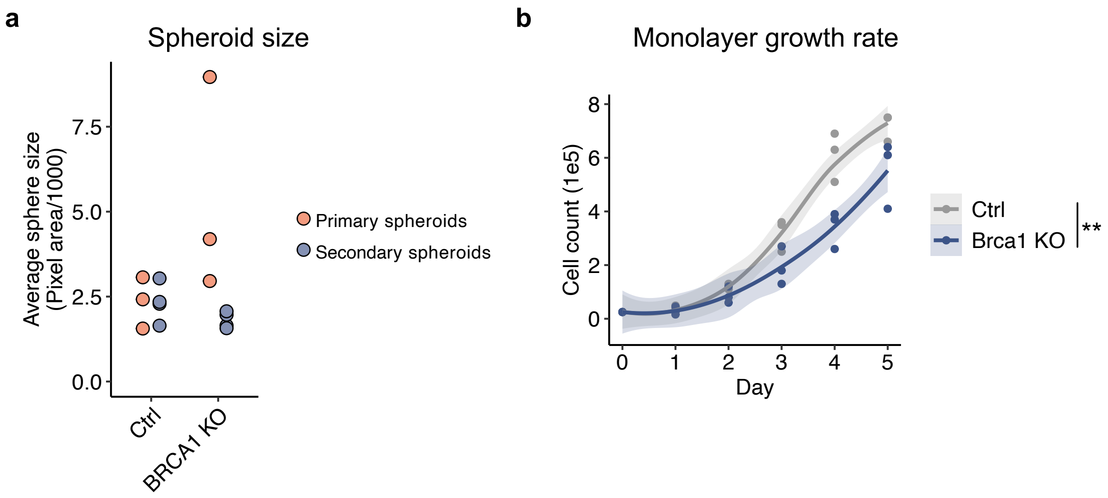

Supplementary Figure 7. a, Relative sizes of spheroids from control and BRCA1 KO mOSE (n=3). Each point on the plot represents the average size of spheroids from 4 fields of view for 4 separate wells. **b,** Proliferation rates of control and BRCA1 KO mOSE (n=3). Each point represents cell counts of an individual replicate and the trend line represents a LOESS regression for each condition with the shaded area representing the 95% confidence interval. ** $p < 0.01$, linear regression on counts from days 2-5.
